# A fully human IgG1 antibody targeting connexin 32 extracellular domain blocks CMTX1 hemichannel dysfunction in an in vitro model

**DOI:** 10.1186/s12964-024-01969-0

**Published:** 2024-12-05

**Authors:** Abraham Tettey-Matey, Viola Donati, Chiara Cimmino, Chiara Di Pietro, Damiano Buratto, Mariateresa Panarelli, Alberto Reale, Arianna Calistri, Maria Vittoria Fornaini, Ruhong Zhou, Guang Yang, Francesco Zonta, Daniela Marazziti, Fabio Mammano

**Affiliations:** 1 CNR Institute of Biochemistry and Cell Biology, Monterotondo, Rome, 00015 Italy; 2 Department of Biomedical Sciences, University of Padua, Padua, 35131 Italy; 3 CNR Institute of Endocrinology and Experimental Oncology “G. Salvatore”, Naples, 80131 Italy; 4 Institute of Quantitative Biology, College of Life Science, Zhejiang University, Hangzhou, Zhejiang 310058 P. R. China; 5 Department of Physics and Astronomy “G. Galilei”, University of Padua, Padua, 35131 Italy; 6 Department of Molecular Medicine, University of Padua, Padua, 35131 Italy; 7 Shanghai Institute for Advanced Immunochemical Studies, ShanghaiTech University, Shanghai, 201210 P. R. China; 8 Department of Biosciences and Bioinformatics, School of Science, Xi’an Jiaotong-Liverpool University, Suzhou, 215123 P. R. China; 9 Present Address, CNR Institute of Biophysics, Genoa, 16149 Italy; 10 Present Address: Interdisciplinary Research Centre On Biomaterials, University of Naples Federico II, Naples, 80125 Italy

**Keywords:** Charcot–Marie–Tooth diseases, Connexons, Cx32, Ca^2+^ uptake, Dye uptake, ATP release, Monoclonal antibodies, Molecular dynamics

## Abstract

Connexins (Cxs) are fundamental in cell–cell communication, functioning as gap junction channels (GJCs) that facilitate solute exchange between adjacent cells and as hemichannels (HCs) that mediate solute exchange between the cytoplasm and the extracellular environment. Mutations in the *GJB1* gene, which encodes Cx32, lead to X-linked Charcot-Marie-Tooth type 1 (CMTX1), a rare hereditary demyelinating disorder of the peripheral nervous system (PNS) without an effective cure or treatment. In Schwann cells, Cx32 HCs are thought to play a role in myelination by enhancing intracellular and intercellular Ca^2+^ signaling, which is crucial for proper PNS myelination. Single-point mutations (p.S85C, p.D178Y, p.F235C) generate pathological Cx32 HCs characterized by increased permeability (“leaky”) or excessive activity (“hyperactive”).

We investigated the effects of abEC1.1-hIgG1, a fully human immunoglobulin G1 (hIgG1) monoclonal antibody, on wild-type (WT) and mutant Cx32D178Y HCs. Using HeLa DH cells conditionally co-expressing Cx and a genetically encoded Ca^2+^ biosensor (GCaMP6s), we demonstrated that mutant HCs facilitated 58% greater Ca^2+^ uptake in response to elevated extracellular Ca^2+^ concentrations ([Ca^2+^]_ex_) compared to WT HCs. abEC1.1-hIgG1 dose-dependently inhibited Ca^2+^ uptake, achieving a 50% inhibitory concentration (EC_50_) of ~ 10 nM for WT HCs and ~ 80 nM for mutant HCs. Additionally, the antibody suppressed DAPI uptake and ATP release. An atomistic computational model revealed that serine 56 (S56) of the antibody interacts with aspartate 178 (D178) of WT Cx32 HCs, contributing to binding affinity. Despite the p.D178Y mutation weakening this interaction, the antibody maintained binding to the mutant HC epitope at sub-micromolar concentrations.

In conclusion, our study shows that abEC1.1-hIgG1 effectively inhibits both WT and mutant Cx32 HCs, highlighting its potential as a therapeutic approach for CMTX1. These findings expand the antibody’s applicability for treating diseases associated with Cx HCs and inform the rational design of next-generation antibodies with enhanced affinity and efficacy against mutant HCs.

**Supplementary Information:**

The online version contains supplementary material available at 10.1186/s12964-024-01969-0.

## Abstract (plain language summary)

Connexins (Cxs) are proteins essential for communication between cells, forming gap junction channels (GJCs) that connect neighboring cells and hemichannels (HCs) that link the inside of cells to their surroundings. Mutations in the *GJB1* gene, which makes Cx32, cause X-linked Charcot-Marie-Tooth type 1 (CMTX1), a rare nerve disorder that currently has no cure. Our research studied the effects of a specially designed antibody, abEC1.1-hIgG1, on both normal and mutated Cx32 HCs using a well-established cell model for studying these channels. We discovered that the antibody significantly reduced the movement of calcium ions (Ca^2+^), which are vital for nerve health, in both normal and mutated channels. Further analysis showed that the antibody’s success might be due to how it binds to certain areas of the channels. Our findings suggest that abEC1.1-hIgG1 could be developed into a treatment for CMTX1 and similar conditions. Moreover, this research could help in creating even more effective antibodies for targeting faulty hemichannels.

**Supplementary Information:**

The online version contains supplementary material available at 10.1186/s12964-024-01969-0.

## Background

Hereditary demyelinating diseases of the peripheral nervous system (PNS), collectively known as Charcot–Marie–Tooth (CMT) diseases, are rare yet among the most common genetic disorders, affecting approximately 1 in 2500 individuals [1]. Currently, there are no treatments available to cure or slow disease progression [2]. X-linked Charcot-Marie-Tooth disease type 1 (CMT1X, MIM #304040) is a dominant subtype, accounting for about 17% of all CMT cases [3]. Patients typically experience progressive muscle weakness, amyotrophy, and sensory abnormalities, especially in the distal extremities [4–6].

CMT1X arises from over 450 known mutations, including missense, frameshift, deletion, and nonsense mutations in the *GJB1* gene, which encodes the protein connexin (Cx) 32 (Cx32) [7, 8]. Cxs form hexameric membrane structures called connexons or hemichannels (HCs), which dock to form gap junction channels (GJCs) between neighboring cells [9]. GJCs enable direct cytoplasmic communication, while undocked HCs mediate ion and molecule exchange with the extracellular environment [10].

In Schwann cells, Cx32 GJCs are crucial for connecting different layers of the myelin sheath, facilitating the diffusion of ions like K^+^ and essential metabolites [11]. As the majority of Cx32 mutations implicated in CMTX1 are loss-of-function [12, 13], the hypothesis is that reduced permeability of mutant GJCs to signaling molecules, such as cyclic adenosine monophosphate (cAMP), disrupts myelin homeostasis and contributes to CMT1X neuropathy [14, 15].

However, CMTX1 is also linked to certain Cx32 gain-of-function mutations (e.g., p.S85C, p.D178Y, and p.F235C) which affect the HCs [16–18]. Cx HCs typically remain closed under physiological conditions, opening transiently only in response to specific stimuli, such as changes in extracellular or cytosolic Ca^2+^ levels [19, 20] to allow the passage of ions and small molecules, influencing cell signaling [21, 22]. ATP released through open HCs can activate purinergic receptors (e.g., P2X and P2Y receptors) on neighboring cells [23, 24], triggering signaling pathways that may promote neuroinflammatory processes [25–27], which can worsen nerve damage and hinder regeneration in hereditary neuropathies [28, 29].

This study evaluates an antibody targeting an extracellular domain shared by Cx26, Cx30, and Cx32 HCs. This antibody has shown nanomolar sensitivity and proven efficacy in inhibiting HCs in previous studies [30–34]. Here, we extend these findings by examining the antibody’s effects on Ca^2+^ influx, dye uptake, and ATP release in cells conditionally expressing wild-type (WT) or p.D178Y mutant Cx32, using the genetically encoded Ca^2+^ indicator GCaMP6s [33, 35]. Our results propose this antibody as a promising candidate for treating CMT1X patients with leaky or hyperactive HCs and offer insights for designing improved therapeutic antibodies.

## Methods

### Viruses

For conditional expression of the Cx of interest and cytosolic GCaMP6s, we followed the methods of Nardin et al. [33] and Tettey-Matey et al. [36]. Briefly, we modified a mammalian expression, lentivirus (LV), Tet-On [37], destination vector with a puromycin selection marker (pCW57.1, a gift from David Root; Addgene plasmid # 41393; http://n2t.net/addgene:41393; RRID: Addgene_41393; accessed on 01 November 2024). A multiple cloning site (MCS) linked to an Internal Ribosome Entry Site (IRES) and GCaMP6s nucleotide sequences (MCS-IRES-GCaMP6s) was gene-synthesized and inserted between the NheI (GCTAGC) and BamHI (GGATCC) restriction enzyme sites in the pCW57.1 vector to form a new LV transfer vector, which we named pCW57-MSC-IRES-GCaMP6s. Next, the MCS in this vector was used to subclone the coding sequence (CDS) of human Cx32 (*GJB1*, NCBI CCDS ID: CCDS14408.1) or its mutant p.D178Y. We thus obtained two corresponding inducible bicistronic lentiviral plasmids, named pCW57-Cx32-IRES-GCaMP6s [36] and pCW57-Cx32D178Y-IRES-GCaMP6s, respectively, for the simultaneous expression of the Cx of interest and cytosolic GCaMP6s. We deposited these plasmids in Addgene where they have been respectively assigned the following IDs: 216795 and 216796 (https://www.addgene.org/Fabio_Mammano/, accessed on 30 October 2024).

### Establishment of inducible cell pools overexpressing the Cx of interest

HeLa DH cells (Merck, 96112022-DNA-5UG) were cultured at 37 °C in a 5% CO_2_/95% air atmosphere using complete growth medium obtained by supplementing Dulbecco’s Modified Eagle Medium (DMEM, high glucose 4.5 g/L, sodium pyruvate 110 mg/L) with 10% heat-inactivated fetal bovine serum (FBS), 2 mM L-glutamine, and 1% penicillin/streptomycin. The culture medium was replaced every other day.

The pCW57-Cx32-IRES-GCaMP6s and pCW57-Cx32D178Y-IRES-GCaMP6s plasmids were used to produce LV particles by transfecting HEK-293 T cells (American Type Culture Collection, CRL-3216) with Lipofectamine 2000 (Thermo Fisher Scientific, TFS, 11668–027) following a standard protocol [38]. LV-containing supernatant was collected, cleared by filtration, and immediately used to infect HeLa DH cells as detailed in Tettey-Matey et al. [36]. Following puromycin selection of virally transduced HeLa DH cells, the ordinary FBS was replaced with Tetracycline negative FBS collected in South America (Capricorn Scientific, FBS-TET-12B).

### qPCR

Total RNA was extracted with TRIzol Reagent (TFS, 15596,018) from HeLa-Cx32-GCaMP6s and HeLa-Cx32D178Y-GCaMP6s cells plated at a density of 2.5 × 10^5^/ml in 35 mm ∅ Petri dishes and exposed or not exposed to doxycycline (dox, 4 µg/ml; Merck, D1822) for 48 h. Ten (10) µg of the extracted total RNA was digested with DNase I (TFS, 18068015) and purified using Rneasy mini Kit (Qiagen, 74104) according to the manufacturer’s instructions. A NanoDrop spectrophotometer was used to quantify the RNA by measuring the absorbance. Reverse transcription PCR (RT-PCR) was performed from 1 μg of the purified RNA using a high-capacity cDNA kit (TFS, 4368814), according to the manufacturer’s instructions but with a slight modification in which Oligo(dt) (TFS, 18418012) was combined with the cDNA kit random primers at a ratio of 1:5.

qPCR was performed on the cDNA with specific primers (see below). Samples were analyzed at least in triplicate and gene expression levels were estimated by the 2^−∆∆CT^ method [39], using glyceraldehyde-3-phosphate dehydrogenase (GAPDH) as the internal reference gene and SYBR green (SsoAdvanced Universal SYBR green Supermix; BioRad, 1725274) on the ABI 7900HT sequence detection system equipped with the AB1 7900HT SDS software (Applied Biosystems) applying the following amplification cycles: 50 °C, 2 min (1 cycle); 95 °C, 10 min (1 cycle); 95 °C, 15 s; 62 °C, 35 s (40 cycles).

The primers used for these experiments were:

- Cx32 (Cx32 WT and Cx32D178Y): Forward 5′-CGTGAACCGGCATTCTACTG-3′; Reverse 5′-TGGTCATAGCAAACGCTGTTG-3′.
- EMCV-IRES: Forward 5′-AATGTGAGGGCCCGGAAACC-3′; Reverse 5′-ACTCACAACGTGGCACTGGG-3′.
- GCaMP6s: Forward 5′-GGAGGACGGCAACATC-3′; Reverse 5′-GAAAGCCTCTTTAAATTCTGCG-3′.
- GAPDH: Forward 5′-CACCATCTTCCAGGAGCGAG-3′; Reverse 5′-CCTTCTCCATGGTGGTGAAGAC-3′.

### Immunofluorescence

HeLa-Cx32-GCaMP6s and HeLa-Cx32D178Y-GCaMP6s cells (1 × 10^5^) were plated onto round glass coverslips (12 mm ∅) in 35 mm ∅ Petri dishes and exposed to dox (4 μg/ml) or vehicle for 48 h. Cells were fixed with 4% paraformaldehyde (PFA; TFS, 047392.9 M) for 15 min, permeabilized with 0.1% Triton X-100 (TFS, A16046.AE) for 5 min and incubated for 1 h in blocking buffer containing 2% bovine serum albumin (BSA; A4503, Merck) and 0.1% Triton X-100. Samples were then incubated overnight at 4 °C with a commercial anti-Cx32 monoclonal antibody (1:200; TFS, 35–8900) diluted in blocking buffer. The next day, samples were washed three times with phosphate buffered saline (PBS; TFS, 10010023) and incubated with Alexa Fluor 555-conjugated Donkey anti-Mouse IgG (1:800; TFS, A-31570) or Alexa Fluor 488-conjugated Goat anti-Mouse IgG (1:800; TFS, A-11029) for 1 h at room temperature. For co-immunofluorescent staining of GCaMP6s, samples were then incubated overnight at 4 °C with anti-GFP polyclonal antibody (1:200; TFS, A-11122), which recognizes also GCaMP6s, diluted in blocking buffer. The next day, samples were washed three times with PBS and incubated with Alexa Fluor 488-conjugated Donkey anti-Rabbit IgG (1:800; TFS, A-21206) for 1 h at room temperature. Cell nuclei were stained with 4',6-diamidino-2-phenylindole (DAPI; TFS, D1306) (5 µg/ml, 5 min) and coverslips were mounted with ProLong Gold (TFS, P36934).

For experiments with abEC1.1-hIgG1, we prepared a Ca^2+^-free medium (ZCM) containing (in mM): 138 NaCl, 5 KCl, 0.4 NaH_2_PO_4_, 6 D-Glucose, 10 HEPES (all from Merck), pH 7.3. Throughout this article, we refer to ECM as the medium obtained by adding 60 μM CaCl_2_ to ZCM. Live cells exposed to dox (4 μg/ml) for 48 h, were incubated at 37 °C in ECM supplemented with 10 nM abEC1.1-hIgG1 for 1 h. Next, cells were fixed with 4% PFA for 10 min and incubated for 1 h with Alexa Fluor 594-conjugated Goat anti-Human IgG (1:800; Jackson ImmunoResearch, 109–585-170). Samples were then washed three times with PBS between all steps and Cx32 staining was performed as indicated above. Cell nuclei were stained with DAPI (5 µg/ml, 5 min).

Visualization of plasma membrane was performed using MemBrite Fix 640/660 (Biotium, 30097). Briefly, live cells were incubated with a solution of MemBrite probes for 1 min at 37 °C, according to manufacturer’s instruction. After incubation, the excess of MemBrite was washed with HBSS, the cells were fixed, and Cx32 and DAPI staining were performed as indicated above.

Immunofluorescence images were acquired with a TCS SP5 confocal microscope (Leica Microsystems) equipped with a 40 × oil immersion objective (Leica Microsystems, HCX PL Apo, UV optimized, N.A. 1.25, oil). Alexa Fluor 488 and GCaMP6s fluorescence was excited by a 488-nm Argon laser and collected between 500 and 540 nm. Alexa Fluor 555 and Alexa Fluor 594 were excited by a 543-nm HeNe laser and collected between 568 and 680 nm and between 600 and 670 nm, respectively.

Immunofluorescence images were also acquired with an FV1200 confocal microscope (Olympus Corporation) equipped with 60 × oil immersion objective (Olympus Corporation, PLAPON60XOSC, N.A. 1.40) and visualized with the FV10-ASW software (version 4.2; Olympus Corporation). Confocal through-focus image sequences (z-stacks) were collected with steps of 0.5 µm along the optical axis. DAPI fluorescence was excited by a 405 laser and collected between 425 and 475 nm; Alexa Fluor 488 fluorescence was excited by a 488 laser and collected between 500 and 545 nm. Alexa Fluor 555 and Alexa Fluor 594 were excited by a 559-nm laser and their emissions were collected between 575 and 620 nm and between 575 and 675 nm, respectively. MemBrite fluorescence was excited by a 635-nm laser and its emission was collected between 655 and 755 nm.

### Assay of GCaMP6s functionality

Forty-eight hours after dox induction (4 μg/ml), cell-plated coverslips with a live, confluent cell population were transferred to the previously described spinning disk confocal system [40]. Cells were maintained under the microscope objective at 37 °C in a heated chamber (1.5 ml capacity) and perfused with ZCM, supplemented with either 2 mM or 0.2 mM Ca^2+^. Sequences of fluorescence images were acquired at 1.5-s intervals using a cooled sCMOS camera (PCO, EDGE; resolution: 2560 × 2160 pixels, 6.5 μm × 6.5 μm, 4 × binning).

GCaMP6s was excited using a 488 nm diode laser (Cat. No. COMPACT-150G-488-SM, World Star Tech), and fluorescence emission (*F*) was collected through a 535/30 nm band-pass filter (Cat. No. ET535/30 M, Chroma Technology). Images were analyzed offline using ImageJ and MATLAB (R2019a, The MathWorks). GCaMP6s signals, *F*(*t*), where *t* denotes the time of frame acquisition, were calculated as pixel spatial averages from regions of interest (ROIs) drawn around the perimeter of at least 10 cells per field of view (FOV). To correct for back reflections and electronic offsets, background signals from a cell-free ROI were subtracted [41].

### Ca^2+^ uptake

Forty-eight hours after dox induction (4 μg/ml), coverslips were transferred to a heated chamber (1 ml capacity) on an upright SP8-DIVE-STED-FALCON laser scanning confocal fluorescence microscope (Leica Microsystems), equipped with a white laser and a 25 × water immersion objective (HC IRAPO, N.A. 1.00). For control experiments, cells were incubated at 37 °C for 15 min in ECM. For pharmacological studies, cells were pre-incubated for 30 min at 37 °C in ECM containing either 100 μM flufenamic acid (FFA; Merck, F9005) or varying concentrations of abEC1.1-hIgG1, ranging from 0.001 nM to 5 μM.

GCaMP6s was excited at 488 nm, and fluorescence emission was spectrally detected between 500 and 535 nm. Sequences of fluorescence images were acquired at 1.3 s intervals for a 6 min period. Following a 10 s baseline recording, a 2 μl bolus of 1 M CaCl_2_ was delivered at the edge of the field-of-view (FOV) using a 2.5 μm Ø glass capillary connected to a pneumatic pico-pump (SYS-PV830, World Precision Instruments). Three min later, 2 μl of 200 μM ionomycin (Merck, I3909) [42] was administered to elicit maximum fluorescence signals.

Image sequences were analyzed offline using Suite2p [43] and MATLAB (R2019a, The MathWorks). GCaMP6s signals were extracted as pixel spatial averages from ROIs drawn around the perimeter of at least 20 cells per FOV. Baseline fluorescence (*F*_0_) traces (one per ROI) were corrected for photobleaching by fitting with an exponential decay function and subtracting this fit from each trace. Each Δ*F*(*t*) trace, defined as Δ*F*(*t*) = *F*(*t*) – *F*_0_, was normalized to its respective Δ*F*_max_, the maximum Δ*F*(*t*) response evoked by ionomycin.

### DAPI uptake

Forty-eight hours after dox induction, coverslips with cells were transferred to a spinning disk confocal microscope [40] equipped with a 20 × water immersion objective (Olympus, XLUMPlan Fl, N.A. 0.95). For control experiments, cells were pre-incubated for 30 min at 37 °C in ECM, followed by a 5-min incubation with 5 μM DAPI in ECM at room temperature in the dark. For pharmacological experiments, cells were similarly pre-incubated for 30 min at 37 °C in ECM containing either 50 μM FFA or 1 μM abEC1.1-hIgG1, followed by the addition of 5 μM DAPI and a 5-min incubation at room temperature in the dark.

DAPI was excited using a 385 nm LED (M385L3, Thorlabs), and emission was collected through a blue band-pass filter (Semrock, FF01-425/26–25) using a cooled sCMOS camera (PCO, EDGE; resolution 2560 × 2160 pixels, 6.5 μm × 6.5 μm, binning 4). To account for DAPI uptake by dead cells, the first image was subtracted from all subsequent images, and DAPI uptake traces, *F*(*t*), were computed as pixel spatial averages from ROIs drawn around the perimeter of at least 40 cell nuclei per FOV. DAPI uptake rates, *dF*(*t*) / *dt*, were estimated by fitting each *F*(*t*) trace with a straight line through the origin [44, 45].

### ATP release

ATP release from HeLa-Cx32D178Y-GCaMP6s cells was measured using a luciferin/luciferase assay (Promega, GA5010, RealTime-Glo™ Extracellular ATP Assay) with a Varioskan LUX plate reader (TFS, VLB000D0). Cells were seeded in 96-well black plates (Corning, 3603) at a density of 2.5 × 10^4^ cells/well. Forty-eight hours after dox induction, the medium was replaced with 75 μl of phosphate-free solution (PFS) containing 138 mM NaCl, 5 mM KCl, 6 mM D-glucose, 10 mM HEPES, 1 mM MgCl_2_, and 2 mM CaCl_2_, pH 7.3. Next, 25 μl of 4X ATP assay reagent was added to each well.

Cells were incubated at 37 °C for 30 min with or without inhibitors (1 μM abEC1.1-hIgG1, 100 μM FFA, or 200 μM LaCl_3_) in PFS. Baseline luminescence readings were recorded every 30 s, with a 1 s exposure per well. ATP release was stimulated by adding 10 μl of 2 μM 4-Br-A23187 (Merck, 100,107) [19], and luminescence measurements continued for additional 10 min. Data were analyzed using MATLAB, and ATP release was quantified as the area under the baseline-subtracted luminescence curve. Non-induced (–dox) cells were used as negative controls.

### Molecular dynamics simulations

The model of Cx32 was constructed using the Protein Data Bank (PDB) crystal structure 7ZXN [46]. Missing segments of the cytoplasmic loop were added with MODELLER [47]. The disordered C-terminal region was truncated from residue 220 onward, consistent with previously published models [48], as structural studies indicate that GJCs formed by different connexins exhibit similar structures in the extracellular region [9]. We hypothesize that truncating the C-terminal does not affect the predictive accuracy of our model, given that the extracellular loops, critical for antibody docking, remain unchanged.

The CHARMM-GUI membrane builder was employed to insert the protein into a POPC lipid bilayer and to introduce the p.D178Y mutation in each protomer of the HC. The fragment antigen-binding (Fab) region of the abEC1.1-hIgG1 antibody was modeled using the AlphaFold-collab web server [49]. We used the HADDOCK web server to dock the Fab to the Cx32 HC, enforcing interactions between the Fab’s Complementarity Determining Regions (CDRs) and the extracellular loops of Cx32. Two top docking configurations were chosen, and molecular dynamics simulation (MDS) were conducted on both the wild-type (Cx32WT) and mutant (Cx32D178Y) systems.

Each system was solvated with TIP3P water, containing Cl^-^ and K^+^ ions at a concentration of ~ 0.15 M to mimic physiological ionic conditions. The total atom count after solvation was approximately 2.5 × 10^5^. We performed energy minimization followed by pre-equilibration simulations in the NVT and NPT ensembles, applying positional restraints on heavy atoms as described previously [50]. Specifically, a 250 ps NVT simulation was conducted with a 1 fs time step, followed by a 1.6 ns NPT equilibration using a 2 fs time step, both with heavy atom restraints. The final production simulation was run for 100 ns.

MDS and energy minimization were performed using GROMACS [51] with the CHARMM36m force field [52]. Long-range electrostatics were handled using the particle mesh Ewald method [53], with a 12 Å cutoff for Lennard–Jones interactions and a switching function starting at 10 Å. Temperature was maintained at 310 K using the V-rescale thermostat [54] with a 1 ps coupling constant, and pressure was controlled at 1 bar using the Parrinello-Rahman barostat [55] with a 5 ps coupling constant. The LINCS algorithm was used to constrain hydrogen-containing bonds, allowing a 2 fs integration time step.

Of the two binding models, only one remained stable throughout the simulation period, so the other was excluded from further analysis. We studied the electrostatic properties using APBS [56] and visualized the results with PyMOL (Schrödinger L, DeLano W. PyMOL, 2020. Available from: http://www.pymol.org/pymol). Additional figures were generated with VMD and Python [57].

### Free energy calculation

The free energy perturbation (FEP) Hamiltonian replica-exchange (HREX) method was used to quantify the impact of residue 178 on the binding affinity of the abEC1.1 Fab to Cx32. The initial frame for these calculations was selected from the Cx32WT MDS trajectory after the root mean square deviation (RMSD) stabilized, around 70 ns. To enhance computational efficiency, the HC was truncated at the start of the transmembrane helices (TMHs), preserving the extracellular region while applying positional restraints to the TMH residues to keep them fixed in space.

The FEP parameter λ was varied from 0 to 1 to simulate the transformation from the wild-type (WT, D178) to the mutant (Y178) state. We used 32 windows with soft-core potentials, each running in parallel. The PMX toolkit [58, 59] was used to parameterize amino acid mutations within the CHARMM36 force field. Simulations were conducted using GROMACS, saving energy values every 10 ps and attempting exchanges between adjacent windows every 2 ps.

We computed the free energy change for both the complex (ΔGcomplex) and the unbound state (ΔGfree) using Alchemical Analysis [60], and then calculated the binding free energy difference (ΔΔG) using a thermodynamic cycle approach [61]. To ensure statistical robustness, we performed five independent replicas for the FEP calculations. Each state within each replica was simulated for 48 ns, with 1.5 ns per window, resulting in a cumulative simulation time of 2.88 μs (48 ns × 2 states × 5 replicas × 6 connexins).

### Statistics

Statistical analyses were performed using MATLAB (R2019a, The MathWorks). The normality of data distributions was assessed with the Shapiro–Wilk test. For normally distributed data, comparisons of means were conducted using ANOVA, followed by Bonferroni post hoc tests for multiple group comparisons or two-tailed t-tests for comparisons between two groups. For non-normally distributed data, the Kruskal–Wallis test was used, with Dunn-Sidak post hoc tests for multiple group comparisons.

Results are presented as mean values ± standard error of the mean (s.e.m.), unless otherwise specified. Sample sizes (n) for each experimental group are indicated in the figure legends. Statistical significance was defined as *p*-values (p) less than 0.05 and denoted with asterisks in the figures: *, *p* < 0.05; **, 0.005 < *p* < 0.05; ***, *p* < 0.0005.

## Results

### Expression analyses of Cx32, CX32D178Y and GCaMP6s in virally transduced HeLa DH cells

HeLa DH, a human cervix carcinoma cell line, has been widely used for studying GJC formation, intercellular GJC and HC permeability, and sensitivity to pharmacological modulators [45, 62–67]. These cells are known for their stable membrane potentials, robustness under double whole-cell patch-clamp measurements, and low endogenous expression of Cx45 [68]. Prior studies have shown that the abEC1.1 antibody has minimal effects on Cx45 HCs [31].

**Fig. 1:**
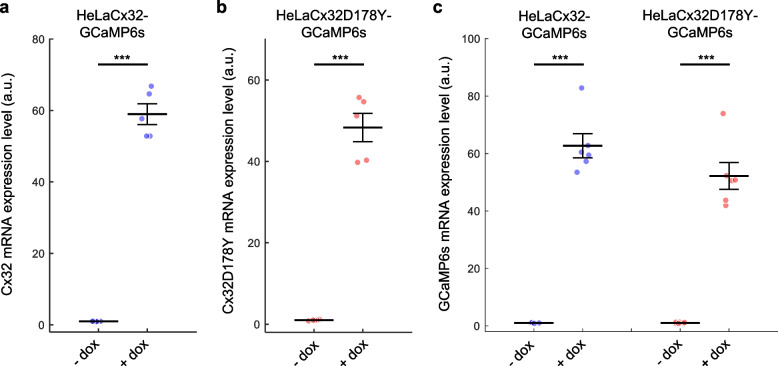
Validation of Tet-On bicistronic lentiviral vectors. Dot plots showing mean ± s.e.m. of mRNA expression levels measured by qPCR in cells exposed to doxycycline for 48 h (+ dox) or not (− dox): **a** Cx32, **b** Cx32D178Y, and **c** GCaMP6s. Asterisks indicate statistical significance (*p*-value, Kruskal–Wallis (KW) test); *n* > 3 independent cell cultures per condition

In our study, we used qPCR assays to analyze the expression of Cx32, Cx32D178Y, and GCaMP6s in inducible pools of HeLa-Cx32-GCaMP6s and Cx32D178Y-GCaMP6s cells. We chose the Tet-On inducible system [37] for Cx expression [33, 36] because constitutive overexpression of hyperactive or leaky HCs is cytotoxic [69, 70]. Upon the addition of dox (4 μg/ml) to the culture medium, Cx mRNA levels increased over 50-fold within 48 h (Fig. 1a, b). A similar increase was observed for GCaMP6s mRNA (Fig. 1c) and IRES mRNA (Fig. S1). The non-zero expression of these mRNAs in the absence of dox is likely due to the minimal basal leakiness of the tetracycline-responsive element (TRE) promoter in the lentiviral construct [71].

**Fig. 2:**
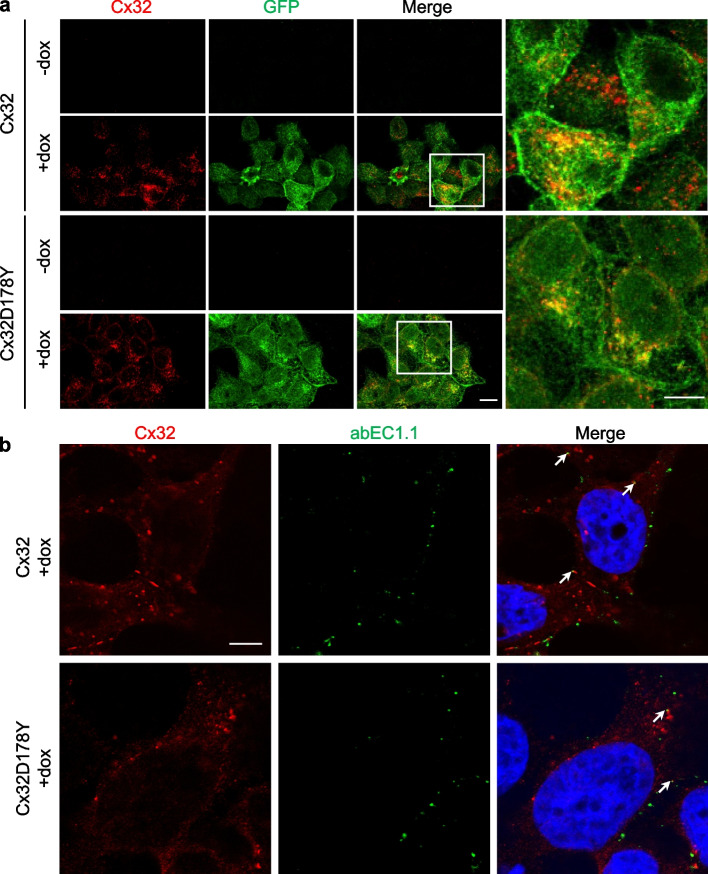
Immunofluorescence characterization of Cx expression. **a** Representative confocal images of fixed and permeabilized cells expressing human WT Cx32 (upper panels) or mutant Cx32D178Y (lower panels), either exposed to doxycycline for 48 h (+ dox) or not exposed (− dox). Cells were labeled with anti-Cx32 (red) and anti-GFP (green) antibodies (the GFP epitope recognized by the antibody is also present in GCaMP6s). Scale bar: 10 µm. Right panels: higher magnification of boxed areas, showing fluorescent puncta at cell–cell contacts; scale bar: 10 µm. **b** Representative confocal images showing immunofluorescence labeling with abEC1.1 and anti-Cx32. Live cells expressing human WT Cx32 (upper panels) or mutant Cx32D178Y (lower panels), exposed to doxycycline for 48 h (+ dox), were treated with 10 nM abEC1.1 antibody. Cells were then fixed (but not permeabilized) and counterstained with a secondary antibody selective for the human Fc domain of IgG (green). After permeabilization, cells were stained with anti-Cx32 (red) and counterstained with DAPI (blue) to visualize nuclei. Scale bar: 5 µm

Protein expression was confirmed by immunofluorescence staining using a well-characterized commercial antibody that recognizes an intracellular epitope in the C-terminus of Cx32 in permeabilized cells [72]. The staining revealed fluorescent puncta distributed throughout the cytoplasm and at cell–cell contacts, indicating the presence of intercellular GJCs (Fig. 2a; Fig. S2), consistent with previous observations of Cx32 and its mutants in HeLa cells [73]. To specifically label HCs at the plasma membrane, live cells were treated with the abEC1.1-hIgG1 antibody, followed by fixation and counterstaining with a fluorescent secondary antibody that recognizes the human Fc domain of the antibody (Fig. 2b) [33].

### Functional assays of the co-expressed Ca^2+^ indicator GCaMP6s

**Fig. 3:**
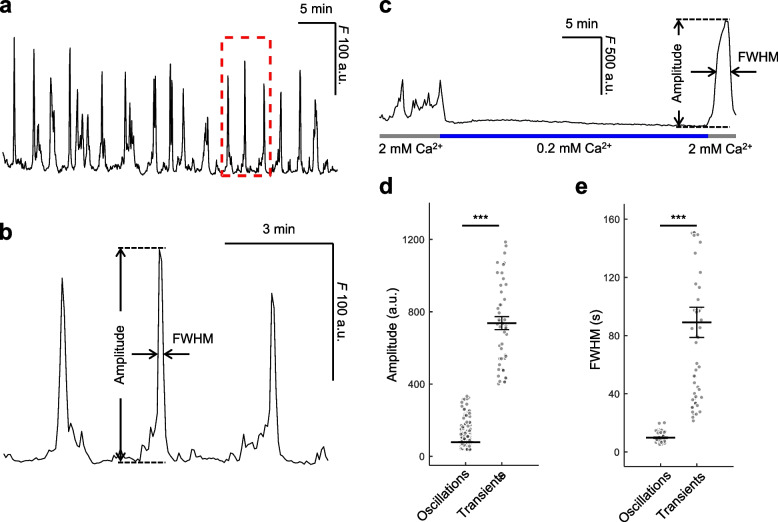
Functional characterization of GCaMP6s expression. **a** Representative trace of GCaMP6s fluorescence (*F*) emission from HeLa-Cx32-GCaMP6s cells during spontaneous [Ca^2+^]_cyt_ oscillations in 2 mM extracellular Ca^2+^ ([Ca^2+^]_ex_). **b** Detail of the oscillations highlighted by the dashed red box in (a) with indication of the parameters used for quantitative data analysis. **c** Effect of reducing [Ca^2+^]_ex_ from 2 mM to 0.2 mM, followed by a return to 2 mM. **d**, **e** Dot plots with mean ± s.e.m. of amplitude (d) and full width at half maximum (FWHM) (e) of spontaneous [Ca^2+^]_cyt_ oscillations in 2 mM [Ca^2+^]ex (left) and [Ca^2+^]_cyt_ transients evoked by increasing [Ca^2+^]_ex_ from 0.2 mM to 2 mM (right). Asterisks indicate statistical significance (*p*-value, Kruskal–Wallis test); *n* = 40 to 60 cells per condition, 5 independent experiments

We conducted Ca^2+^ imaging experiments to confirm the functionality of the co-expressed GCaMP6s indicator (Fig. 3 and Video S1). When cells were maintained in a solution containing 2 mM extracellular Ca^2+^ ([Ca^2+^]_ex_), Cx HCs remained mostly closed [20], resulting in only brief, low-amplitude fluctuation of the GCaMP6s fluorescence signal, *F*(*t*), attributed to spontaneous cytosolic Ca^2+^ ([Ca^2+^]_cyt_) oscillations (Fig. 3a, b) [74]. The amplitudes and inter-peak intervals of these oscillations displayed non-normal frequency distributions (Fig. S3).

Lowering [Ca^2+^]_ex_ to 0.2 mM halted the [Ca^2+^]_cyt_ oscillations within 3 min (Fig. 3c) and opened the HCs [20]. The rapid reintroduction of 2 mM [Ca^2+^]_ex_ triggered a substantial Ca^2+^ influx through the open HCs [33, 75], resulting in pronounced, longer-duration [Ca^2+^]_cyt_ transients (Fig. 3c). The mean amplitude and full width at half maximum (FWHM) of these transients were both 9-fold higher, compared to those of the spontaneous [Ca^2+^]_cyt_ oscillations (Fig. 3d, e). These experiments confirmed two key points: (i) GCaMP6s expressed after dox induction effectively reports [Ca^2+^]_cyt_ fluctuations and (ii) the [Ca^2+^]_cyt_ transients induced by the reintroduction of 2 mM [Ca^2+^]_ex_ are readily distinguishable from the baseline oscillations observed under normal [Ca^2+^]_ex_ conditions.

### Effect abEC1.1-hIgG1 on Ca^2+^ uptake

**Fig. 4:**
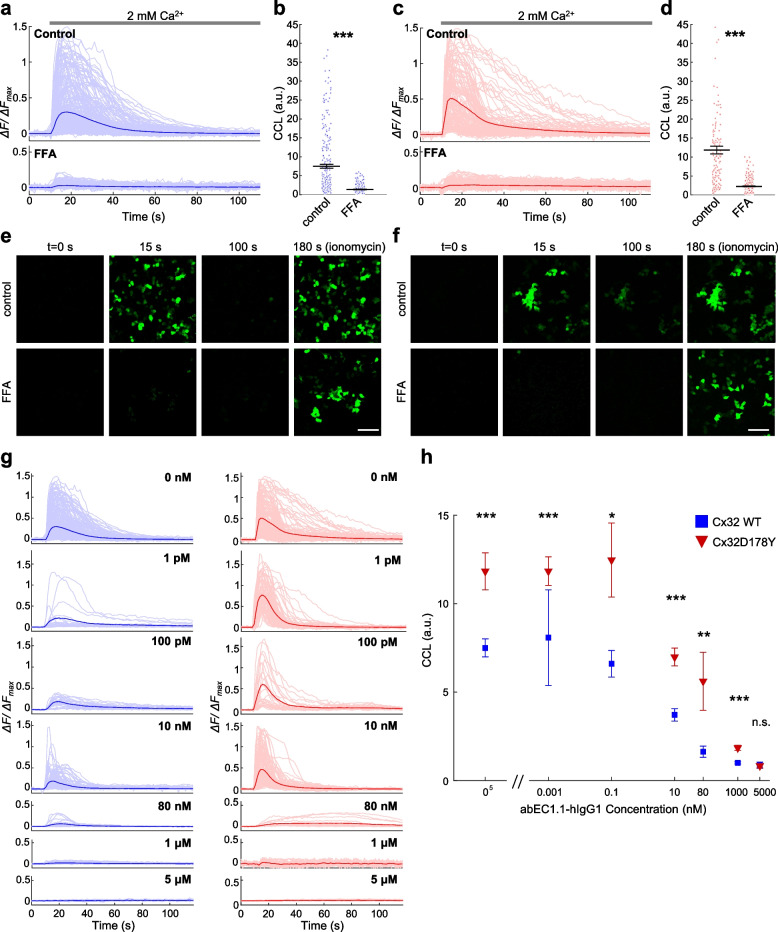
Ca^2+^ uptake through Cx32 and Cx32D178Y HCs. **a**, **c** Mean GCaMP6s Δ*F*/Δ*F*_max_ traces (thick lines) in response to a Ca^2+^ stimulus, overlaid with individual cell responses (light lines) from doxycycline-induced HeLa-Cx32-GCaMP6s (**a**) or HeLa-Cx32D178Y-GCaMP6s (**c**) cell cultures. Cells were maintained in ECM (Control, upper panels; *n* > 140 cells) or ECM containing 100 µM FFA (lower panels; *n* > 260 cells). **b**, **d** Dot plots showing cytosolic Ca^2+^ load (CCL), measured as the area under Δ*F*/Δ*F*_max_ traces from *t* = 0 to *t* = 100 s, with mean ± s.e.m. superimposed. **e**, **f** Representative sequences of fluorescence images taken at the indicated time points (corresponding to the x-axis in panels **a** and **c**, from Video S2-S5); scale bar: 100 µm. **g** Mean GCaMP6s Δ*F*/Δ*F*_max_ traces (thick lines) overlaid with individual cell responses (light lines) from doxycycline-induced HeLa-Cx32-GCaMP6s (left panels, blue) and HeLa-Cx32D178Y-GCaMP6s (right panels, red) cell cultures in ECM supplemented with increasing concentrations of abEC1.1-hIgG1. **h** Dose-dependent effect of abEC1.1-hIgG1 on the CCL induced by Ca^2+^ uptake through Cx32 HCs (blue squares) and Cx32D178Y HCs (red triangles), based on traces in (**g**). Data are presented as mean ± s.e.m. Asterisks indicate statistical significance (*p*-value, Kruskal–Wallis test); *n* > 15 cells per condition

Given the role of deregulated Ca^2+^ signaling in the pathogenesis of CMTX1 [17, 76], we quantified Ca^2+^ uptake through open HCs (Fig. 4 and Video S2-S5) [33, 75]. Pre-incubation with 100 μM FFA, a common Cx channel inhibitor [77], significantly reduced the cytosolic Ca^2+^ load (CCL), measured as the area under the Δ*F*(*t*) curve of GCaMP6s, in both HeLa-Cx32-GCaMP6s and HeLa-Cx32D178Y-GCaMP6s cells (Fig. 4a-f), and this inhibitory effect was reversible (Fig. S4). These experiments confirmed that Ca^2+^ uptake predominantly involved overexpressed HCs. Notably, the CCL in HeLa-Cx32D178Y-GCaMP6s cells was 58% higher than in HeLa-Cx32-GCaMP6s cells (*p* = 1.3 × 10^-6^, KW test), consistent with the notion that the p.D178Y mutation leads to deregulated Ca^2+^ handling by HCs [17].

Next, we evaluated the effect of abEC1.1-hIgG1 on Ca^2+^ uptake (Fig. 4g, h). In HeLa-Cx32-GCaMP6s cells, the antibody began inhibiting Ca^2+^ uptake at 100 pM, with a half-maximal inhibitory concentration (EC_50_) around 10 nM and maximal inhibition achieved at concentrations above 1 μM. In HeLa-Cx32D178Y-GCaMP6s cells, the antibody remained ineffective at concentrations up to 100 pM, showed an EC_50_ around 80 nM, and reached maximal inhibition at concentrations above 5 μM (Fig. 4h).

### Effect abEC1.1-hIgG1 on DAPI uptake and ATP release

To further validate our observations, we performed DAPI uptake experiments, a widely used method to assess Cx HC activity under various conditions [44, 45]. DAPI is a small fluorescent molecule (277 Da) that can permeate most Cx channels and fluoresces intensely upon binding to A-T-rich regions of nuclear DNA, significantly enhancing its quantum yield [78]. Previous studies have shown that DAPI uptake in Cx-expressing HeLa cells is more than 10 times faster compared to parental HeLa cells, with uptake rates correlating closely with HC density [65, 79, 80].

**Fig. 5:**
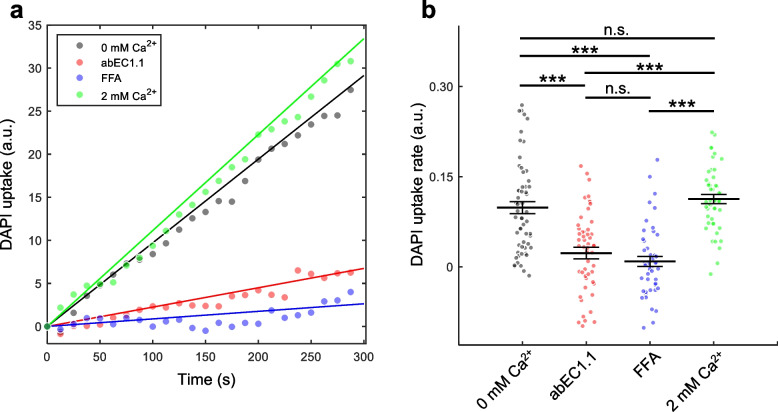
DAPI uptake through Cx32D178Y HCs. **a** Kinetics of DAPI uptake 48 h post-dox induction. The plots represent the time course of the mean fluorescence intensity from ROIs encompassing the nuclei of *n* = 40 to 60 cells per condition, with data fitted using a least-squares linear regression through the origin. Experiments were performed in 0 Ca^2+^ ZCM, with 1 μM abEC1.1-hIgG1 or 50 μM FFA added as indicated. **b** Dot plots showing DAPI uptake rates, with mean ± s.e.m. superimposed. Asterisks indicate statistical significance (*p*-value, Kruskal–Wallis test); *n* = 40 to 60 cells per condition

In our current experiments, we observed that DAPI uptake in HeLa-Cx32D178Y-GCaMP6s cells, induced with dox for 48 h, was not influenced by changes in [Ca^2+^]_ex_. However, a 30 min pre-incubation with either 50 μM FFA or 1 μM abEC1.1-hIgG1 significantly reduced the DAPI uptake rate (Fig. 5). These results further support the hypothesis that (i) the p.D178Y mutation disrupts the normal Ca^2+^ regulation of Cx32 HC activity [17], and (ii) the abEC1.1 blocks Cx32D178Y mutant HCs.

**Fig. 6:**
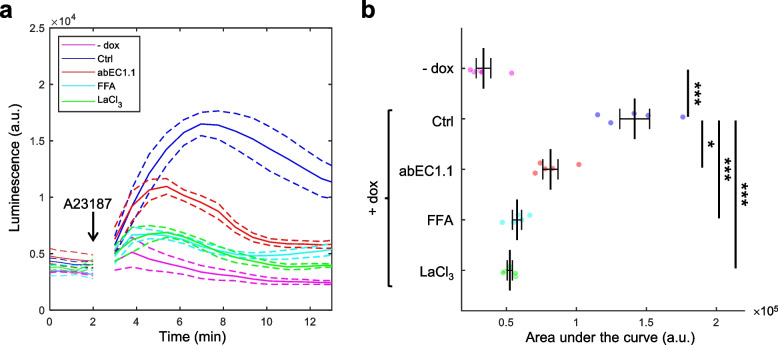
ATP release through Cx32D178Y HCs. **a** Kinetics of ATP release, shown as mean luminescence signals (solid lines) ± s.e.m. (dashed lines) over time. Gaps in the data following the addition of 2 μM 4-Br-A23187 are due to instrumentation limitations. **b** Dot plots representing the quantification of luminescence data from panel (**a**), with mean ± s.e.m. superimposed. Asterisks indicate statistical significance (*p*-value, ANOVA test); *n* = 5 cell cultures per condition

Finally, we also examined ATP release, a key factor in CMTX1 pathogenesis, where aberrant HC activity contributes to disrupted cellular homeostasis [76]. In Cx32-expressing cells, ATP release can be triggered by transient increases in [Ca^2+^]_cyt_, such as with 4-Br-A23187 at concentrations of 1.5–2 μM [19]. Previous studies demonstrated that abEC1.1 inhibits ATP release from mutant Cx30 HCs linked to Clouston syndrome [32]. Here, we used bioluminescence measurements with a luciferin-luciferase kit to monitor ATP release from Cx32D178Y HCs in a 96-well plate format at 37 °C (Fig. 6). Non-induced (− dox) cells exhibited minimal ATP release, while dox-induced cells showed a robust ATP release response, peaking within 5 min of 2 μM 4-Br-A23187 stimulation, at levels 4-fold higher than baseline (Fig. 6a). The ATP release was significantly inhibited by abEC1.1-hIgG1, FFA, and lanthanum chloride (LaCl_3_), a non-specific HC blocker [44, 81]. A statistical comparison of the area under the ATP release curve is shown in Fig. 6b.

### Modeling abEC1.1-hIgG1 interaction with Cx32 and Cx32D178Y HCs

Previous studies demonstrated that other gain-of-function mutations in Cx HCs, such as Cx30 p.A88V, Cx26 p.G45E and Cx26 p.D50N, did not impact the efficacy of the abEC1.1 antibody when administered in a single-chain format (scFv-Fc) [30, 32, 34]. These mutations are located within the central pore of the HC and are distant from the epitope targeted by the antibody [31]. In contrast, our Ca^2+^ uptake experiments suggested that the p.D178Y mutation, located in the second extracellular loop of Cx32, reduces the affinity of the antibody for Cx32 HCs. This reduction in affinity may be due to the involvement of residue D178 in the interaction with the antibody [31].

**Fig. 7:**
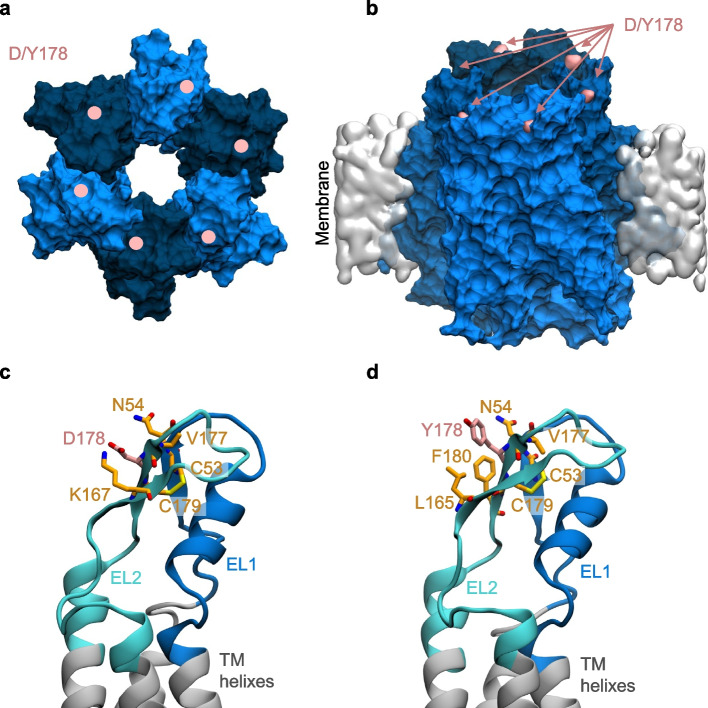
Atomistic models of WT and D178Y mutant Cx32 HCs. **a** Top view of a Cx32 HC from the extracellular perspective, rendered in surface mode. The six connexin subunits are shown in alternating dark and light blue. The positions of the D/Y178 residues in each connexin are indicated by pink dots. **b** Side view of a Cx32 HC embedded in the lipid membrane, with D/Y178 residues highlighted in pink. **c**, **d** Close-up views of the residues interacting with D178 (**c**) and Y178 (**d**) within a connexin. Both panels depict the extracellular loops (EL1, EL2) and parts of the transmembrane (TM) helices, showing residues within 3 Å of D/Y178. D/Y178 residues are colored in pink, while the surrounding interacting residues are shown in orange

**Fig. 8:**
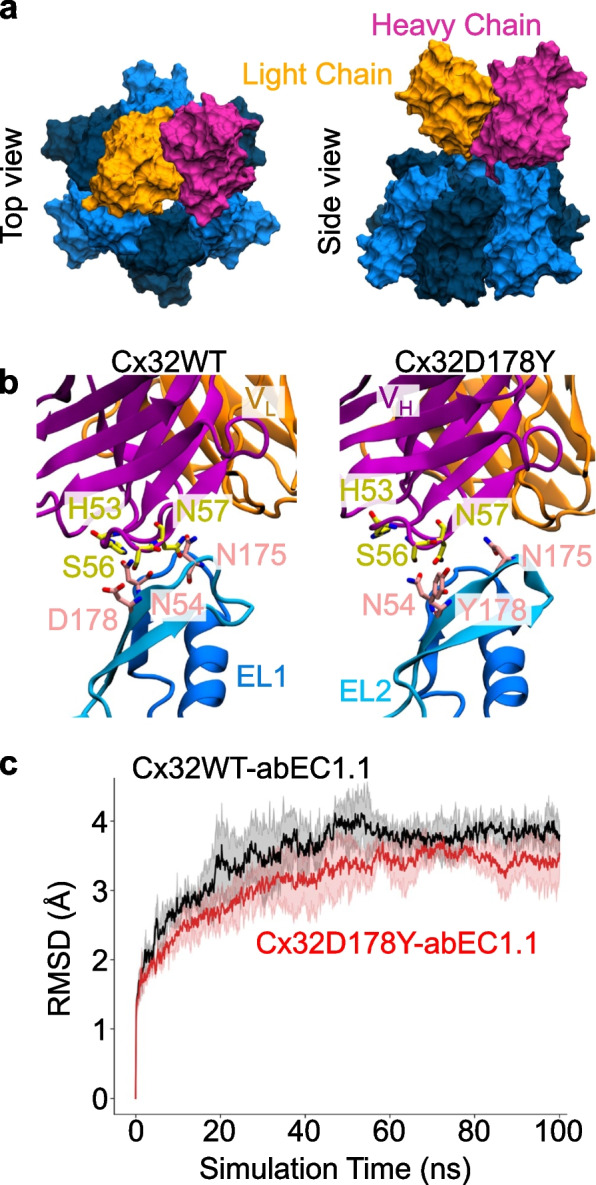
Model of abEC1.1-hIgG1 Fab bound to WT or mutant Cx32 HC. **a** Final configuration of the HC-Fab complex after 100 ns of MDS, shown from the extracellular view (top) and side view (bottom). Proteins are rendered in surface mode, with connexins depicted in alternating dark and light blue, and the Fab heavy chain and light chain colored in pink and orange, respectively. For clarity, only the extracellular loops and portions of the transmembrane (TM) helices are shown for the HCs. **b** Close-up of the interaction between the D/Y178 residue and the Fab. Only one of the six D/Y178 residues directly interacts with the S56 residue of the Fab. Proteins are displayed in cartoon representation. **c** Comparison of the RMSD during the MDS for the WT and D178Y complexes. Results are presented as the mean (solid line) with standard deviation (shaded area) from three independent replicas

To explore this hypothesis and understand the inhibitory mechanism of the antibody, we constructed a structural model of the Cx32 HC (Fig. 7 and Fig. S5) bound to the Fab region of abEC1.1-hIgG1, following methods used previously for modeling abEC1.1(scFv-Fc) [30]. Docking simulations using the HADDOCK web server generated multiple binding modes, which were generally consistent with one another. Among the top-scoring configurations, we selected a model closely resembling the binding of abEC1.1_scFv-Fc to Cx26 (Fig. 8). The stability of this binding mode was tested using a 100 ns MDS, and the results were compared with a parallel simulation in which all six D178 residues were mutated to tyrosine (Y).

The final conformations of the WT and mutant HC-Fab complexes were highly similar (Fig. 8a, b). Both systems equilibrated within 30 ns, and the RMSD from the initial structure stabilized around 3.5 Å. A second docking model was evaluated but produced unstable trajectories during the 100 ns MDS (Fig. S6) and was therefore discarded.

To quantify the impact of the p.D178Y mutation, we analyzed the percentage of time key amino acids in the HC were in close contact (< 3 Å) with the Fab residues throughout the MDS. Data analysis revealed that only one of the six D178 residues maintained a stable interaction (97% of the simulation time) with serine 56 (S56) on the Fab. The other D178 residues contributed minimally to the interaction. A detailed interaction analysis (Table S1 and S2) indicated that the mutation from aspartate to tyrosine at position 178 significantly reduced contact frequency with the antibody. Specifically, the interaction rate of residue 178 with the antibody decreased from 79% in the WT to just 19% in the mutant.

### In silico estimate of binding affinity between abEC1.1 Fab and Cx32 or Cx32D178Y HCs

Analyzing the MDS trajectory alone is often insufficient to fully understand the impact of mutations on the binding affinity between an antibody and its target [82]. Even though molecular simulations can track atomic positions throughout the simulation, they may not clearly reveal the true energetic effects of a mutation. To address this, we performed a direct calculation of the contribution of the residue at position 178 to the binding energy of the Cx32 HC-Fab complex using the Free Energy Perturbation method with Hamiltonian Replica Exchange (FEP-HREX) to improve convergence [83]. The FEP method is a well-established approach for accurately estimating relative binding affinities in diverse chemical and biological systems, including antigen–antibody interactions [84].

**Fig. 9:**
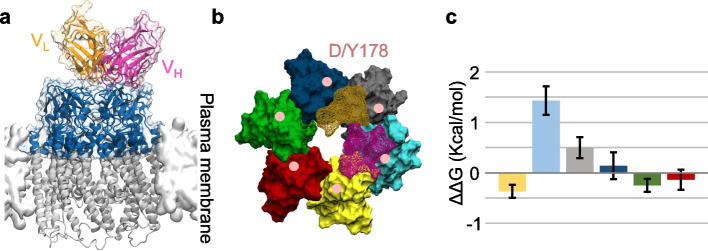
Binding energy difference (∆∆G) induced by the p.D178Y mutation. **a** Side view of the Cx32 HC bound to the Fab region of abEC1.1-hIgG1. Only the colored portion of the HC was used for FEP calculations. The Fab’s heavy chain and light chain are shown in purple and orange, respectively (VL = light chain variable domain, VH = heavy chain variable domain). **b** Top view of the HC model. The HC is displayed in surface mode, with each Cx protomer rendered in a different color. Fab residues that interact with the HC (within a distance of < 4 Å) are depicted in wire-frame surface mode, with the heavy chain in purple and the light chain in orange. Pink dots mark the location of the D178Y mutation. **c** Binding energy differences (∆∆G) resulting from the p.D178Y mutation for each Cx protomer, presented as mean ± s.e.m. The color of each bar corresponds to the respective connexin protomer colors shown in panel **b**

In our model, only one of the six D178 residues maintains close contact with the Fab. However, we considered the possibility that the p.D178Y mutation might also indirectly influence the local structure of the extracellular loops, affecting antibody binding. To test this, we mutated each of the six D178 residues individually to tyrosine (Y) and calculated their respective contributions to the overall change in binding affinity using FEP (Fig. 9a, b).

Our results showed that the D178 residue in direct contact with the antibody accounted for the largest change in binding free energy, causing a ∆∆G increase of approximately 1.43 kcal/mol (cyan bar in Fig. 9c). Mutations at two neighboring Cx protomers produced smaller effects, with ∆∆G values of 0.50 kcal/mol and -0.37 kcal/mol (gray and yellow bars in Fig. 9c), even though these residues did not directly interact with the Fab. This suggests that the mutations subtly altered the arrangement of nearby amino acids involved in binding. The remaining three Cx protomers showed no significant contributions to the Fab docking.

Since the residues at position 178 on different Cx protomers do not interact with each other, the overall binding energy difference can be estimated by summing the individual contributions. The total ∆∆G was calculated to be 1.32 kcal/mol, corresponding to an approximate 10-fold decrease in binding affinity. This result is in excellent agreement with our experimental observations.

## Discussion

Our study demonstrates that abEC1.1-hIgG1 effectively inhibits Ca^2+^ influx, DAPI uptake and ATP release through mutant Cx32D178Y HCs implicated in CMTX1. Importantly, our data reveal that while the p.D178Y mutation reduces the antibody’s affinity, it does not eliminate its inhibitory effect, suggesting that even challenging mutations can be targeted with substantial efficacy.

One of the most compelling aspects of our findings is the mechanistic insight into how the antibody interacts with mutant HCs. MDS and in silico binding studies revealed that the substitution of aspartic acid with tyrosine at position 178 disrupts a critical interaction with the S56 residue of the antibody’s Fab region. This disruption affects both the electrostatic and steric properties at the binding interface, providing a molecular explanation for the observed reduction in antibody affinity. However, the overall structural integrity of the extracellular loops allows the antibody to maintain a meaningful level of inhibition.

These observations emphasize the importance of understanding specific residue interactions, suggesting therapeutic potential also for other leaky of hyperactive Cx32 mutants, such as p.S85C and p.F235C, implicated in CMTX1 pathogenesis. Since these mutations are located far from the extracellular antibody-binding domain, the antibody would likely inhibit these mutants similarly, without interference, potentially expanding its therapeutic application across multiple CMTX1 genotypes. This hypothesis is supported by earlier studies of other gain-of-function mutations in Cx HCs far from the antibody’s target epitope (such as Cx26 p.G45E, Cx26 p.D50N and Cx30 p.A88V), which did not alter the antibody’s effectiveness [30, 32, 35]. Prior work from our lab and others has shown that Cx-targeting antibodies can restore cellular homeostasis by mitigating HC overactivity in mouse models of Keratitis-Ichthyosis-Deafness (KID) syndrome and Clouston syndrome [32, 34, 85]. The pathological mechanisms in HC-related conditions often share similarities [70], suggesting that therapeutic strategies like abEC1.1-hIgG1 could have broader applications [86]. These preclinical successes underscore the potential of HC-specific antibodies to address the root causes of hereditary diseases characterized by connexin dysfunction.

Nevertheless, we must acknowledge the limitations of our study. First, our work relies heavily on in vitro assays. Although these experiments are highly informative and controlled, they do not fully capture the complexity of peripheral nervous system (PNS) pathology in CMTX1. The absence of in vivo data is a significant limitation that must be addressed in future research. Animal models of CMTX1, such as transgenic mice expressing human Cx32 mutations [87, 88], will be essential for assessing the antibody’s efficacy under physiological conditions.

Furthermore, our use of truncated Cx32 models for computational studies, while necessary for simplification, may overlook interactions involving the full-length protein, including the cytoplasmic domains that are absent in our simulations. Advances in artificial intelligence (AI) leveraging the groundbreaking work of 2024 Nobel laureates Demis Hassabis and John Jumper can now accelerate antibody development [89]. These technologies will almost certainly expedite the creation of antibodies tailored to target hyperactive HCs while minimizing off-target effects [90].

Our study also opens avenues for exploring combination therapies. Treatments such as adeno-associated virus (AAV)-mediated antibody gene delivery [34] with novel capsid variants targeting the CNS and PNS [91] could offer a comprehensive approach to CMTX1 management [92]. Given the genetic heterogeneity of CMTX1 and related neuropathies, such a multifaceted approach could provide personalized and durable therapeutic outcomes. However, this therapeutic strategy would require extensive preclinical validation to ensure safety and efficacy. As the International Mouse Phenotyping Consortium (IMPC) continues to develop a broader allelic series for Cx mutations [93], testing abEC1.1-hIgG1 and other rationally designed antibodies across these models will be crucial for establishing their efficacy against a range of CMTX1 variants.

## Conclusion

Our study underscores the therapeutic potential of abEC1.1-hIgG1 for treating CMTX1 by effectively reducing aberrant Ca^2+^ influx and ATP release through hyperactive Cx32 HCs. The antibody’s demonstrated efficacy against both wild-type and mutant Cx32 HCs indicates its broader applicability for multiple CMTX1 variants and related hereditary neuropathies. Moreover, our findings highlight the promise of antibodies as targeted interventions that address the underlying molecular mechanisms of hereditary diseases caused by Cx mutations [86]. This is further supported by the successful preclinical use of abEC1.1 in managing pathological HC activity in conditions such as KID and Clouston syndromes [32, 34, 85]. As antibody engineering and delivery technologies continue to advance, abEC1.1-hIgG1 represents a significant step toward developing effective, mutation-specific therapies for a range of hereditary disorders linked to connexin dysfunction.

## Supplementary Information

Additional file 1.

## Data Availability

No datasets were generated or analysed during the current study.

## Abbreviations

AAV: Adeno-associated virus
ATP: Adenosine triphosphate
cAMP: Cyclic adenosine monophosphate
Ca^2+^: Ionized calcium
[Ca^2+^]_cyt_: Cytosolic free Ca^2+^ concentration
[Ca^2+^]_ex_: Extracellular free Ca^2+^ concentration
CCL: Cytosolic Ca^2+^ load (area under the curve)
CDR: Complementarity determining region
CDS: Coding sequence
CMT: Charcot–Marie–Tooth
CMTX1: X-linked Charcot Marie Tooth type 1
CNS: Central nervous system
Cx: Connexin
DAPI: 4′,6-Diamidino-2-phenylindole
ECM: Extracellular medium containing 60 μM Ca^2+^
Fab: (Antibody) fragment antigen-binding (region)
FEP: Free energy perturbation
FFA: Flufenamic acid
GJC: Gap junction channel
HC: Hemichannel
HREX: Hamiltonian replica-exchange
IRES: Internal Ribosome Entry Site
hIgG1: Human immunoglobulin G1
IMPC: International Mouse Phenotyping Consortium
MCS: Multiple cloning site
MDS: Molecular dynamics simulation
n: Sample size
p: *p*-Value
PDB: Protein data bank
PFS: Phosphate-free solution
PNS: Peripheral nervous system
RMSD: Root mean square distance
scFv-Fc: Single chain fragment variable-fragment crystallizable (antibody format)
s.e.m.: Standard error of the mean
TMH: Transmembrane helix
TRE: Tetracyclin responsive element
WT: Wild type
ZCM: Extracellular medium containing 0 mM Ca^2+^ (also known as Ca^2+^-free medium)
