# Correction: A fully human IgG1 antibody targeting connexin 32 extracellular domain blocks CMTX1 hemichannel dysfunction in an in vitro model

**DOI:** 10.1186/s12964-024-01988-x

**Published:** 2024-12-12

**Authors:** Abraham Tettey-Matey, Viola Donati, Chiara Cimmino, Chiara Di Pietro, Damiano Buratto, Mariateresa Panarelli, Alberto Reale, Arianna Calistri, Maria Vittoria Fornaini, Ruhong Zhou, Guang Yang, Francesco Zonta, Daniela Marazziti, Fabio Mammano

**Affiliations:** 1 CNR Institute of Biochemistry and Cell Biology, Monterotondo, Rome, 00015 Italy; 2 Department of Biomedical Sciences, University of Padua, Padua, 35131 Italy; 3 CNR Institute of Endocrinology and Experimental Oncology “G. Salvatore”, Naples, 80131 Italy; 4 Institute of Quantitative Biology, College of Life Science, Zhejiang University, Hangzhou, Zhejiang 310058 P. R. China; 5 Department of Physics and Astronomy “G. Galilei”, University of Padua, Padua, 35131 Italy; 6 Department of Molecular Medicine, University of Padua, Padua, 35131 Italy; 7 Shanghai Institute for Advanced Immunochemical Studies, ShanghaiTech University, Shanghai, 201210 P. R. China; 8 Department of Biosciences and Bioinformatics, School of Science, Xi’an Jiaotong-Liverpool University, Suzhou, 215123 P. R. China; 9 Present Address: CNR Institute of Biophysics, Genoa, 16149 Italy; 10 Present Address: Interdisciplinary Research Centre On Biomaterials, University of Naples Federico II, Naples, 80125 Italy

**Correction: Cell Commun Signal 22, 589 (2024)**

10.1186/s12964-024-01969-0.

Following the publication of the original article [1], the authors noticed an error in affiliation 8.

The incorrect affiliation 8 is: Department of Biological Sciences, School of Science, Xi’an Jiaotong-Liverpool University, Suzhou 215123, P. R. China.

The correct affiliation 8 is: Department of Biosciences and Bioinformatics, School of Science, Xi’an Jiaotong-Liverpool University, Suzhou 215123, P. R. China.

Further to this, in the section “**Free energy calculation**”, first paragraph, the word ‘distance’ should be replaced with ‘deviation’, so the sentence reads: “The initial frame for these calculations was selected from the Cx32WT MDS trajectory after the root mean square deviation (RMSD) stabilized, around 70 ns.”

The original article [1] has been corrected.
