# Supplementary material for: A fully human IgG1 antibody targeting connexin 32 extracellular domain blocks CMTX1 hemichannel dysfunction in an in vitro model
Source: Cell Commun Signal. 2024 Dec 5;22:589. doi: 10.1186/s12964-024-01969-0 (PMC11619691; doi:10.1186/s12964-024-01969-0)
Supplement: Supplementary file 1 — Additional file 1. [file 12964_2024_1969_MOESM1_ESM.zip › Supplementary Information_2_ESM.docx]

**Supplementary Information**

A Fully Human IgG1 Antibody Targeting Connexin 32 Extracellular Domain Blocks CMTX1 Hemichannel Dysfunction in an vitro model

Abraham Tettey-Matey^1,†,¶^, Viola Donati^1,2, †,^, Chiara Cimmino^3,†,Γ^, Chiara Di Pietro^1†^, Damiano Buratto^4,†^, Mariateresa Panarelli^6^, Alberto Reale^6^, Arianna Calistri^6^, Maria Vittoria Fornaini^6^, Ruhong Zhou^4^, Guang Yang^7^, Francesco Zonta^8,^*, Daniela Marazziti^1,^*, and Fabio Mammano ^5,1,^*

**
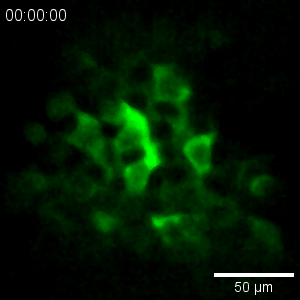
**

**Video S1.** A representative sequence of fluorescence images from the functional assays of the co-expressed Ca²⁺ indicator GCaMP6s in doxycycline-induced HeLa-Cx32-GCaMP6s cells maintained in ZCM supplemented with 2 mM Ca²⁺. Time stamps: hrs:min:sec.


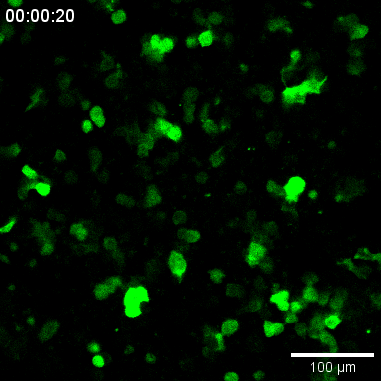


**Video S2.** A representative sequence of fluorescence images from Ca²⁺ uptake experiments through Cx32 HCs in doxycycline-induced HeLa-Cx32-GCaMP6s cells maintained in ECM. Following a 10 s baseline, a bolus of CaCl_2_ (2 μl, 1 M) was added to the ECM, and after ~3 min a bolus of ionomycin (2 μl, 200 μM) was administered to elicit maximum fluorescence signals. Time stamps: hrs:min:sec.


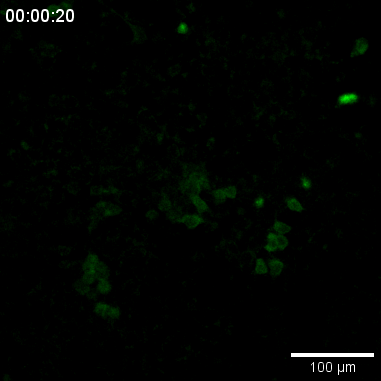


**Video S3.** A representative sequence of fluorescence images from Ca^2+^ uptake experiments through Cx32 HCs in doxycycline-induced HeLa-Cx32-GCaMP6s cells pre-incubated and maintained in ECM supplemented with 100 μM FFA. Following a 10 s baseline, a bolus of CaCl_2_ (2 μl, 1 M) was added to the ECM, and after ~3 min a bolus of ionomycin (2 μl, 200 μM) was administered to elicit maximum fluorescence signals. Time stamps: hrs:min:sec.


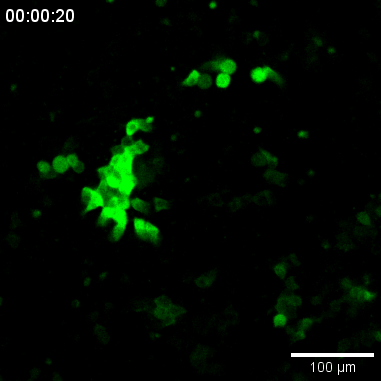


**Video S4.** A representative sequence of fluorescence images from Ca^2+^ uptake experiments through Cx32D178Y HCs in doxycycline-induced HeLa-Cx32D178Y-GCaMP6s cells maintained in ECM. Following a 10 s baseline, a bolus of CaCl_2_ (2 μl, 1 M) was added to the ECM, and after ~3 min a bolus of ionomycin (2 μl, 200 μM) was administered to elicit maximum fluorescence signals. Time stamps: hrs:min:sec.


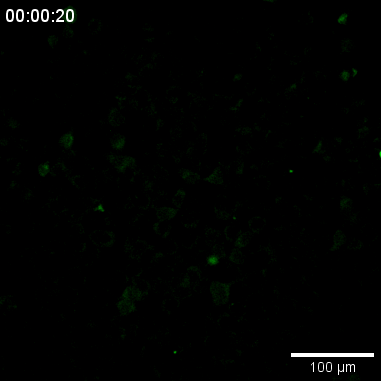


**Video S5.** A representative sequence of fluorescence images from Ca^2+^ uptake experiments through Cx32D178Y HCs in doxycycline-induced HeLa-Cx32D178Y-GCaMP6s cells pre-incubated and maintained in ECM supplemented with 100 μM FFA. Following a 10 s baseline, a bolus of CaCl_2_ (2 μl, 1 M) was added to the ECM, and after ~3 min a bolus of ionomycin (2 μl, 200 μM) was administered to elicit maximum fluorescence signals. Time stamps: hrs:min:sec.
